# Chaotic Pulse-Shaping Filter Based on Root-Raised-Cosine Division

**DOI:** 10.3390/e25010136

**Published:** 2023-01-10

**Authors:** Xiaosi Tian, Zulin Wang, Qin Huang

**Affiliations:** School of Electronic and Information Engineering, Beihang University, Beijing 100191, China

**Keywords:** chaotic filter, wireless communication, ISI, dynamical system, Lyapunov exponent

## Abstract

Chaotic baseband wireless communication system (CBWCS) suffers bit error rate (BER) degradation due to their intrinsic intersymbol interference (ISI). To this end, an ISI-free chaotic filter based on root-raised-cosine (RRC) division is constructed to generate a chaotic signal. A wireless communication system using this chaotic signal as a baseband waveform is proposed. The chaotic property is proved by the corresponding new hybrid dynamical system with topological conjugation to symbolic sequences and a positive Lyapunov exponent. Simulation results show that under a single-path channel and multi-path channel, the proposed method outperforms CBWCS in both BER performance and computational complexity.

## 1. Introduction

Chaos, as a deterministic system, exhibits quasi-random and unpredictable behavior from a simple but nonlinear rule. A number of characteristics of low-frequency broadband, sensitivity to initial values, and impulsive autocorrelation give chaos a good application prospect in the field of chaos-based physical-layer security [1,2,3,4,5]. In practice, chaos communication has been included in some communication standards. Many chaos-based modulation schemes have been proposed as promising solutions for the ultra-wide band radio standards in wireless personal area networks [6]. Frequency-modulated differential chaos shift keying (FM-DCSK) has been adopted as the standard IEEE 802.15.6 [7]. Furthermore, chaos has been applied in high-speed long-distance communication over a commercial fiber-optic channel [8]. However, for the high-rate narrowband wireless communication systems, such as Wi-Fi and cellular networks, there are few practical applications of chaos-based communications in the available literature. The main reasons are as follows. First, the coherent chaotic communication is difficult to be realized due to the difficulty of chaos synchronization between transmitters and receivers in multi-path time-varying channels. Second, the existing chaos-based wireless communications require complicated hardware and software support. Third, non-coherent chaotic communication, such as DCSK, requires a broadband spectrum, which is not applicable to wireless channels with limited-frequency bands. To overcome those problems, the chaotic transmission method in the universal narrowband wireless communication system framework needs to be explored.

Chaotic baseband wireless communication system (CBWCS), first proposed in [9], constructs chaotic signal as baseband waveform by using a special basis function as a pulse-shaping filter. A second-order linear system with an analytic solution provides the expression of the filter [10]. Its infinitely long and exponentially increasing precursor provides determinism and a positive Lyapunov exponent. Due to the invariance of the Lyapunov exponent over the wireless channel [11], CBWCS has excellent performance when communicating in the presence of bounded noise. At the receiver, instead of complex chaotic synchronization steps, a simple matched-filter structure corresponding to the pulse-shaping filter is used to maximize the signal-to-noise ratio (SNR) [12]. Thus, the whole scheme is easily implementable in the universal wireless communication platform [9,13,14,15].

Furthermore, the chaotic pulse is applicable to all scenarios where root-raised-cosine (RRC) can be used as a pulse-shaping filter. Ref. [16] used raised-cosine (RC) windowing to mitigate the effect of intercarrier interference in an orthogonal frequency division multiplexing system. Ref. [17] compared the performance of RC and RRC in filter bank multicarrier/offset quadrature amplitude modulation. In [18], RRC is used in a band-limited spatial modulation massive multi-input multi-output system. Ref. [19] presented an analysis of the influence of RC and RRC and their roll-off factors over the additive white Gaussian noise (AWGN) and the Rician fading channel on communication quality. A detailed analysis of generalized frequency division multiplexing performance under Hoyt-q, Weibull-v and log-normal shadowing fading channels with RC as a pulse-shape filter is represented in [20]. In all communication scenarios mentioned above with RC or RRC as a pulse-shaping filter, chaotic pulses can be applied.

However, the aforementioned pulse-shaping filter does not satisfy the Nyquist intersymbol interference (ISI) criterion. The presence of intrinsic ISI degrades system performance. Refs. [21,22] designed a threshold sequence in the symbol detection process to mitigate ISI from the filter itself and multi-path propagation. However, the threshold structure is complex, which requires high computational complexity in threshold calculation.

In this paper, we design a novel chaotic pulse-shaping filter to generate an ISI-free chaotic baseband waveform. Taking RRC as a starting point, an unequal division method is proposed, which can not only retain the ISI-free property of RC but also produce asymmetric pulses that provide the oscillatory nature of the chaotic signal. Firstly, the RRC pulse is divided into three parts, with the time when the amplitude first decays to zero as a division point. The first two parts are combined to construct an asymmetric pulse. The overall response of those pulses can still hit zero at symbol sampling instants, which meets the Nyquist ISI criteria, such as RC. Then, taking the proposed pulse as an analytical solution of differential equations, a new hybrid dynamical system is constructed with an infinitely long and exponentially increasing precursor. Its chaotic property is proved by the positive maximal Lyapunov exponent.

Implementing the proposed pulse as a shaping filter, a novel chaotic baseband wireless communication system is constructed. At the receiver, a mirrored copy of the transmitted pulse shaping as a matched filter is introduced, which avoids the complicated chaos synchronization between the transmitter and receiver. A corresponding threshold is calculated to relieve the ISI caused by multi-path propagation. Simulation results show that the bit error rate (BER) performance of the proposed method is very close to the lower bound of BER in a single-path AWGN channel. Under a multi-path channel, for BER = $10^{-3}$, the proposed method outperforms CBWCS by more than 2 dB when the threshold is zero. By setting optimal and suboptimal thresholds, the proposed method maintains a satisfactory BER, while the computational complexity of the threshold calculation is reduced by 52.5%.

The rest of this paper is organized as follows. Section 2 briefly reviews the RC and RRC filter. The division method and new dynamical system are presented in Section 3. In Section 4, a novel chaotic wireless communication system is constructed, and its performance is analyzed. Finally, conclusions are drawn in Section 5.

## 2. Backgrounds

In wireless communication systems, to maintain the minimum ISI, the overall responses of the transmit filter, channel response and receive filter have to satisfy Nyquist ISI criterion. The RC pulse has become the de facto standard, which has the impulse response

(1) $$h_{rc}\left(t\right)=\left\{\begin{array}{ccc} \frac{\pi }{4}\frac{\sin\left(\frac{\pi }{2\beta }\right)}{\frac{\pi }{2\beta }}, & \; & t=\pm \frac{T_{s}}{2\beta },\\ \frac{\sin\left(\frac{\pi t}{T_{s}}\right)}{\frac{\pi t}{T_{s}}}\frac{\cos\left(\frac{\beta \pi t}{T_{s}}\right)}{1-{\left(\frac{2\beta t}{T_{s}}\right)}^{2}}, & \; & \;\mathrm{otherwise}\;, \end{array}\right.$$

where β (0<β≤1) is the roll-off factor, and $T_{s}$ is the reciprocal of symbol rate. Its frequency-domain description is a piecewise-defined function, given by:

(2) $$\begin{array}{c} H_{rc}\left(f\right)=\left\{\begin{array}{ccc} 1, & \; & \left|f\right|\leq \frac{1-\beta }{2T_{s}},\\ \frac{1}{2}\left[1+\cos\left(\frac{\pi T_{s}}{\beta }\left[\left|f\right|-\frac{1-\beta }{2T_{s}}\right]\right)\right], & \; & \frac{1-\beta }{2T_{s}}<\left|f\right|\leq \frac{1+\beta }{2T_{s}},\\ 0, & \; & \;\mathrm{otherwise}\;. \end{array}\right. \end{array}$$

The total effective filter in the transmission system is the convolution of the transmit filter and receive filter and the convolution in time domain is equal to the multiplication in the frequency domain. Taking the square root of the RC filter in the frequency domain, we obtain the so-called RRC filter, which has the frequency response $H_{rrc}\left(f\right)$ as follows:

(3) $$H_{rc}\left(f\right)=H_{rrc}\left(f\right)H_{rrc}\left(f\right),$$

or:

(4) $$H_{rrc}\left(f\right)=\sqrt{|H_{rc}\left(f\right)|}.$$

The impulse response of RRC is written as:

(5) $$\begin{array}{c} h_{rrc}\left(t\right)=\left\{\begin{array}{ccc} \frac{1}{T_{s}}\left(1+\beta (\frac{4}{\pi }-1)\right), & \; & t=0,\\ \frac{\beta }{T_{s}\sqrt{2}}\left[\left(1+\frac{2}{\pi }\right)\sin\left(\frac{\pi }{4\beta }\right)+\left(1-\frac{2}{\pi }\right)\cos\left(\frac{\pi }{4\beta }\right)\right], & \; & t=\pm \frac{T_{s}}{4\beta },\\ \frac{1}{1-{\left(\frac{4\beta t}{T_{s}}\right)}^{2}}\left[\cos\frac{(1+\beta )\pi t}{T_{s}}+\frac{T_{s}}{4\beta t}\sin\frac{(1-\beta )\pi t}{T_{s}}\right], & \; & otherwise. \end{array}\right. \end{array}$$

Unlike RC, the impulse response of RRC is not zero at intervals of $\pm T_{s}$. However, the combination of two RRC filters forms a RC pulse, which is zero at intervals of $\pm T_{s}$. Furthermore, since the filter is real-valued and symmetric, RRC is its own matched filter.

## 3. RRC-Divided Chaotic Filter

In this section, we first propose a RRC division scheme to obtain two asymmetric pulses. Their convolution result is compared with the standard RC waveform. Taking the impulse response of the novel filter as the basis function, one new hybrid dynamical system is derived. Its topological conjugation to a symbolic sequence and a positive Lyapunov exponent are proven.

### 3.1. RRC-Division Scheme

**Figure 1:**
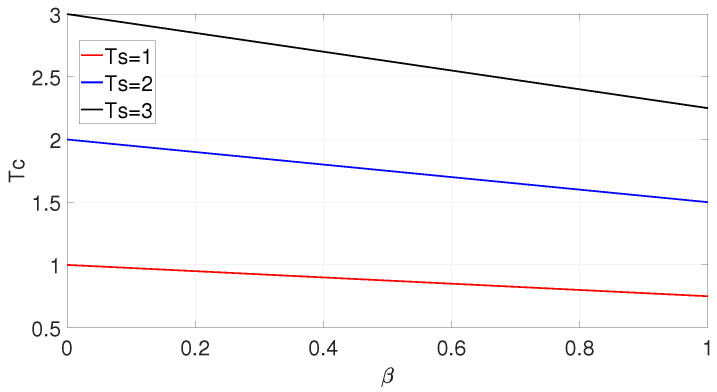
Positive $T_{c}$ with various β and $T_{s}$.

Since the RRC waveform attenuates symmetrically with t=0 as the center, taking the first zero crossing time $T_{c}$ as a division point (one positive and one negative), the waveform can be divided into three parts. $T_{c}$ can be calculated by:

(6) $$\frac{1}{1-{\left(\frac{4\beta T_{c}}{T_{s}}\right)}^{2}}\left[\cos\frac{\left(1+\beta \right)\pi T_{c}}{T_{s}}+\frac{T_{s}}{4\beta T_{c}}\sin\frac{\left(1-\beta \right)\pi T_{c}}{T_{s}}\right]=0,$$

which is a function of the roll-off factor β and symbol time $T_{s}$. Since Equation (6) has infinite solutions, there is no exact analytic solution for zero crossing time. Using fsolve in MATLAB to solve the nonlinear equation, a positive $T_{c}$ with various β and $T_{s}$ can be obtained, as shown in Figure 1.

We can see that when $T_{s}$ is constant, $T_{c}$ decreases linearly with the growth of β. Thus, $T_{c}$ is a linear function of $T_{s}$. The intercept is $T_{s}$, and the slope of the function is −0.25β. Thus, $T_{c}$ can be derived as:

(7) $$T_{c}=-0.25\beta T_{s}+T_{s}.$$

Since RRC is symmetric at t=0, taking $-T_{c}$ and $T_{c}$ as division points, the waveform of RRC can be divided into three parts. By multiplying the first part by two and then combining it with the second part, we can obtain an asymmetric basis function p(t). By combining the second part with the third part by two, another asymmetric basis function g(t) is derived.

From the points discussed above, the impulse responses of p(t) and g(t) are obtained as:

(8) $$\begin{array}{c} p\left(t\right)=\left\{\begin{array}{ccc} 2h_{rrc}\left(t\right), & \; & t<-T_{c},\\ h_{rrc}\left(t\right),\; & \; & -T_{c}\leq t\leq T_{c}, \end{array}\right. \end{array}$$

(9) $$\begin{array}{c} g\left(t\right)=p\left(-t\right)=\left\{\begin{array}{ccc} h_{rrc}\left(t\right),\; & \; & -T_{c}\leq t\leq T_{c},\\ 2h_{rrc}\left(t\right), & \; & t>T_{c}, \end{array}\right. \end{array}$$

where $h_{rcc}$ is shown in Equation (5).

**Figure 2:**
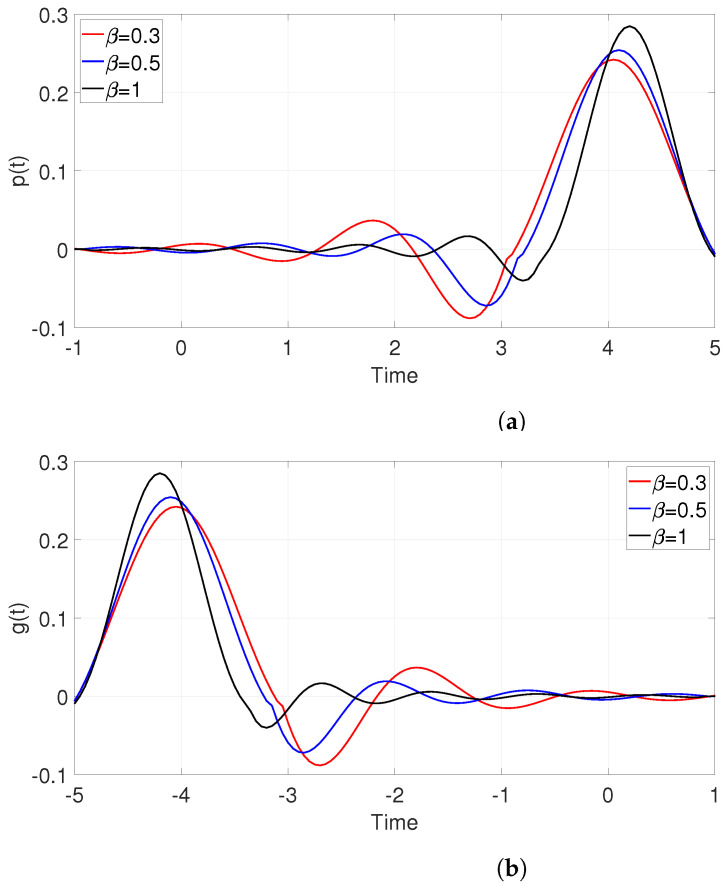
Basis function of (**a**): p(t) and (**b**): g(t) for β=0.3,0.5,1 and $T_{s}=1$.

**Figure 3:**
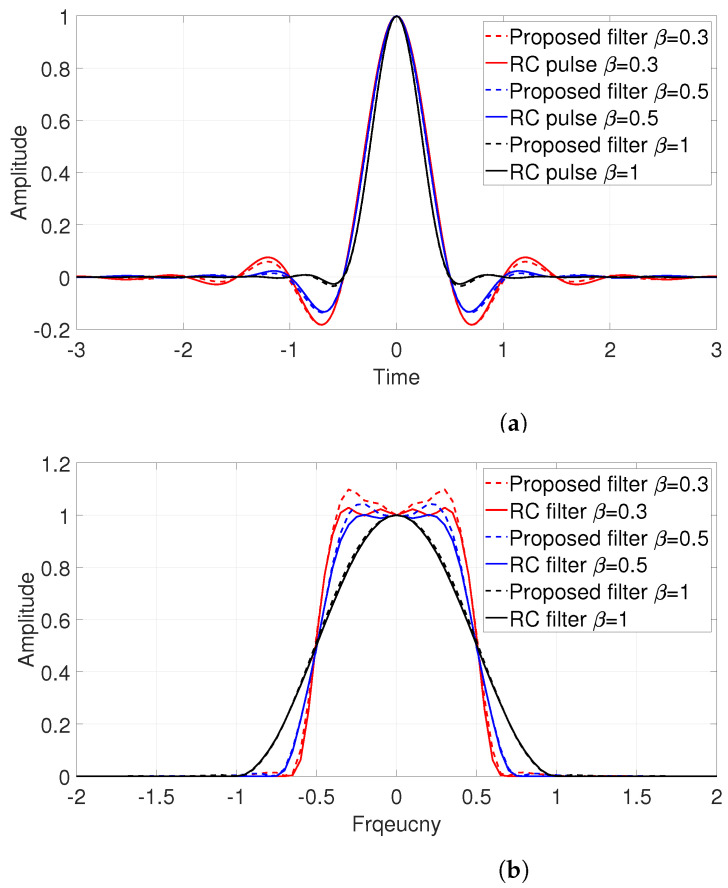
Comparison of convolution of p(t) and g(t) and raised-cosine (RC) pulse in (**a**) time domain and (**b**) frequency domain for β=0.3,0.5, 1 and $T_{s}=1$.

Figure 2 gives the basis function of Equations (8) and (9) with different β. It can be seen that a large value of β results in fast sidelobe decay rates. Figure 3 compares the results of convolution of those two pulses and a theoretical RC pulse. Figure 3a shows the waveforms of two pulses in the time domain, and Figure 3b shows the corresponding frequency domain responses. Due to the approximation error of the division point, the frequency response is slightly jittery compared with the standard RC waveform. However, the ability to provide a zero crossing at the optimal sampling point of other pulse intervals is presented.

All the pulses satisfying the vestigial sideband criterion, namely, that the pulse spectrum has odd symmetry about the corresponding ideally bandlimited spectrum band edge, will be ISI-free. There are an infinite number of such pulses corresponding to different vestigial sidebands. The proposed division method is applicable to all those pulses with the implementation of zero crossing points.

### 3.2. Hybrid Dynamical System

Taking Equation (8) as the basis function and carrying out a linear convolution with the random bi-polar information symbol s(t) as the analytical solution of the differential equation [23], we construct a new hybrid dynamical system to discuss the dynamic behavior of the generated chaotic signal, as follows:

(10) $$\ddot{x}\left(t\right)-2B\dot{x}\left(t\right)+\left[B^{2}+\pi^{2}{\left(\beta +1\right)}^{2}\right]x\left(t\right)=As\left(t\right),$$

(11) $$t\ddot{x}\left(t\right)-2Bt\dot{x}\left(t\right)+2\dot{x}\left(t\right)+\left[B^{2}+\pi^{2}{\left(\beta -1\right)}^{2}\right]tx\left(t\right)-2Bx\left(t\right)=s\left(t\right),$$

where x(t), $\dot{x}\left(t\right)$ and $\ddot{x}\left(t\right)$ are the encoded signal and the first and second derivative of x(t), respectively. In this dynamical system, $T_{s}$ is normalized to 1. The new hybrid dynamical system contains two linear differential equations. A chaotic signal can be obtained by summing the solutions of these two differential equations. Parameters *A* and *B* have two sets of values according to $\left|t\right|=\frac{T_{s}}{4\beta }$. For $\left|t\right|>\frac{T_{s}}{4\beta }$, $A=168e^{-3.755\beta }$, B=4.8(t<0),−4.8(t>0), and for $\left|t\right|\leq \frac{T_{s}}{4\beta }$, $A=6.5\times 10^{-4}e^{13.63\beta }$, B=−0.1(t<0),0.1(t>0).

The generated chaotic signal has an exact analytic solution given by:

(12) $$x\left(t\right)=\sum_{m=-\infty }^{\infty }s\left(m\right)\cdot p\left(t-m\right).$$

#### 3.2.1. Topological Conjugation

For a communication system, the information symbols can be recovered from the received signal only if the encoding baseband waveform and the symbol sequence are topologically conjugated [9,24]. By defining $\phi_{m}\left(t\right)=s\left(m\right)\cdot p\left(t-m\right)$, we have:

(13) $$x\left(t\right)=\sum_{m=-\infty }^{\infty }s\left(m\right)\cdot p\left(t-m\right)=\sum_{m=-\infty }^{\infty }\phi_{m}\left(t\right).$$

The sufficient conditions for the topological conjugation between the chaotic waveform x(t) and driving symbols s(m)(m=−∞,…,+∞) are [9]:

Conditions: For all a,b∈A, fixed constants G,H>0, and α,γ>1:

1. $\left|\phi_{a}\left(t\right)\right|<H\alpha^{-|t|}$;
2. $\phi_{a}\left(t\right)\neq \phi_{b}\left(t\right)$ almost everywhere for a≠b;
3. $max_{a,b}\left|\phi_{a}\left(t\right)-\phi_{b}\left(t\right)\right|<G\cdot \gamma^{-|t|}$, where G∈R;
4. $\min_{a\neq b}\int_{0\leq t<1}\left|\phi_{a}\left(t\right)-\phi_{b}\left(t\right)\right|dt>\int_{t<0}\max_{a,b}|\phi_{a}\left(t\right)-\phi_{b}\left(t\right)|dt$.

Under the assumptions of s(m)∈{+1,−1}, the above conditions are satisfied. The proof is as follows:

For Condition 1, since the solution of differential Equation (11) x(t) has an exponential decay, this condition is satisfied.

For Condition 2, when k≠j, $\phi_{k}\left(t\right)\neq \phi_{j}\left(t\right)$ is satisfied, because when $s_{a}\in \left\{+1,-1\right\}$ and $s_{b}\in \left\{+1,-1\right\}$, $\left|p(t-a)\right|\neq \left|p(t-b)\right|$.

For Condition 3, we have $\max_{a,b}\left|\phi_{a}\left(t\right)-\phi_{b}\left(t\right)\right|=2\left|p\left(t\right)\right|<2A\times 2^{-|t|}$. Then, the condition is satisfied.

For Condition 4, we have:

$\min_{a\neq b}\int_{|t|<1/2}\left|\phi_{a}\left(t\right)-\phi_{b}\left(t\right)\right|dt=2\int_{|t|<1/2}\left|p\left(t\right)\right|dt$,

$\max_{a\neq b}\int_{|t|>1/2}\left|\phi_{a}\left(t\right)-\phi_{b}\left(t\right)\right|dt=2\int_{|t|>1/2}\left|p\left(t\right)\right|dt$.

**Figure 4:**
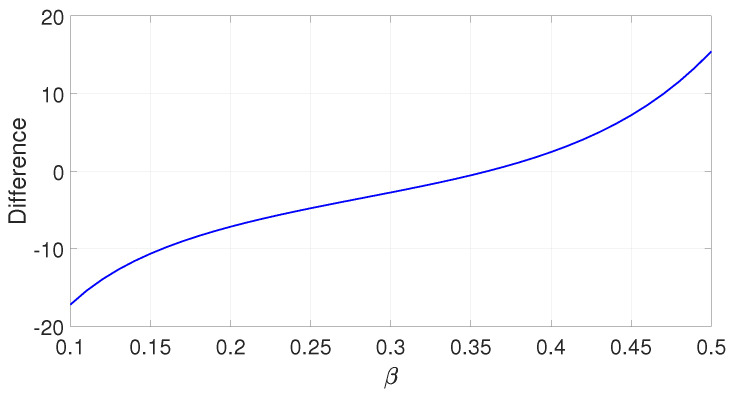
Curve of $\int_{|t|<1/2}|h\left(t\right)|dt-\int_{|t|>1/2}\left|h\left(t\right)\right|dt$.

The curve of $\int_{|t|<1/2}|p\left(t\right)|dt-\int_{|t|>1/2}\left|p\left(t\right)\right|dt$ versus β is given in Figure 4, from which, this condition is satisfied if β>0.3602.

Because of topological conjugation, we can not only construct a chaotic waveform from a symbol sequence, but we can also reversely recover a symbol sequence from a chaotic waveform.

#### 3.2.2. Dynamic Behavior

**Figure 5:**
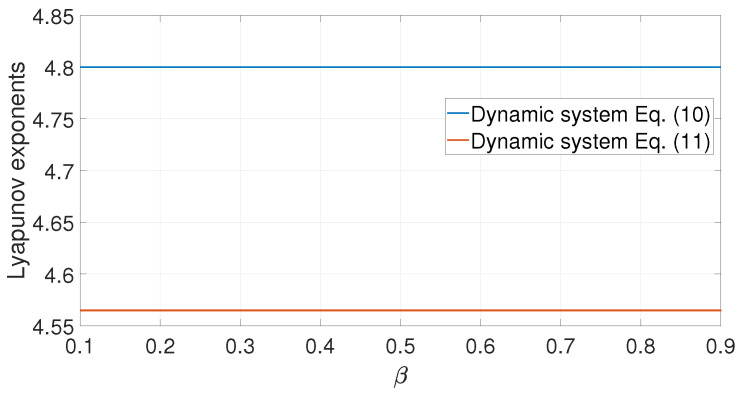
Lyapunov exponents of dynamical system Equations (10) and (11).

The Lyapunov exponent is an important index to judge whether a dynamical system is chaotic. Figure 5 shows the calculation of the maximum Lyapunov exponents of the system of Equations (10) and (11), which are 4.8 and 4.5648, respectively. The Lyapunov exponent remains unchanged when parameter *B* is invariant. Furthermore, the Lyapunov dimension of the chaotic signal is unchanged by the wireless channel [11], which means that the baseband waveform can be transmitted in a physical media that linearly filters signals.

## 4. Wireless Communication System Using Chaotic Filter

Applying the proposed chaotic filter as a pulse-shaping filter and matched filter, a novel chaotic baseband wireless communication system is constructed. The effects of different wireless channels on the system are discussed. The BER performance and computational complexity of different methods are compared.

### 4.1. Transmitter and Receiver

**Figure 6:**
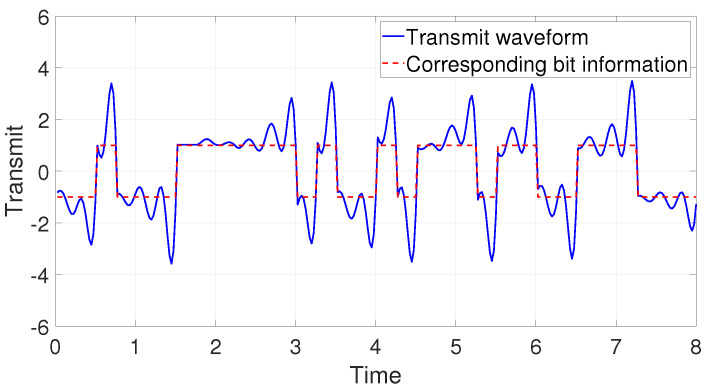
Encoding chaotic waveform and corresponding bit information.

In modern digital communication systems, binary information symbols are usually mapped into a predefined constellation using constellation mapping, such as multi-phase shift keying (MPSK) and multi-stage quadrature amplitude modulation (MQAM). In this paper, binary-phase shift keying (BPSK) is taken as an example, and binary information is mapped to obtain bipolar symbols $s_{m}\in \left\{+1,-1\right\}$. After being oversampled, mapped symbols are then encoded by an RRC-based chaotic pulse-shaping filter p(t) to generate the baseband waveform x(t). Different roll-off factors of the shaping filter can be selected according to different needs. The encoding chaotic baseband wireless waveform at the transmitter with β=0.5 and the corresponding bit information are shown in Figure 6 with a blue solid line and red dotted line, respectively.

The received signal passing through the wireless channel can be written as:

(14) r(t)=h(t)∗x(t)+w(t),

where ‘∗’ denotes convolution, w(t) is AWGN with $w∼CN\left(0,\sigma^{2}\right),\;\mathrm{and}\;\sigma^{2}=N_{0}$. h(t) is the impulse response of the wireless channel, which can be modeled as:

(15) $$h\left(t\right)=\sum_{l=0}^{L-1}\alpha_{l}\delta \left(t-\tau_{l}\right),$$

where $\alpha_{l}$ and $\tau_{l}$ are the attenuation and propagation delay corresponding to path *l*, respectively, and δ(·) is the Dirac delta function. Assume that delay (l=0,1,…,L−1) satisfies $0=\tau_{0}<\tau_{1}<\ldots <\tau_{L-1}$, and channel fading $\alpha_{l}$ is modeled as a negative exponential decay $\alpha_{l}=e^{-\gamma \tau_{l}}$, where γ is the damping coefficient. Equation (15) is a statistical average channel model [25] for a practical wireless communication channel, and it is considered for the theoretical performance analysis.

At the receiver, the matched filter g(t) expressed in Equation (9) is used to maximize SNR. The filter output y(t) is:

(16) $$\begin{array}{cc} y(t) & =r(t)*g(t)\\ \; & =s(t)*p(t)*h(t)*g(t)+W(t), \end{array}$$

where W(t)=w(t)∗g(t) is the filtered noise. If w(t) is AWGN with a zero mean, then W(t) is also Gaussian noise with a zero mean [26].

After sampling y(t) by n=⌊t⌋, a symbol decision algorithm is implemented as:

(17) $$s\left(n\right)=\left\{\begin{array}{cc} 1, & \;\mathrm{if}\;\left(y(n)\geq \theta (n)\right),\\ -1, & \;\mathrm{if}\;\left(y(n)<\theta (n)\right), \end{array}\right.$$

where θ(n) is the threshold to carry out the symbol decision process. The threshold calculation algorithm for the proposed method is described as follows.

### 4.2. Threshold Calculation

The total impulse response of the pulse-shaping filter, wireless channel and matched filter can be described as:

(18) $$\begin{array}{cc} R(t) & =p(t)*h(t)*g(t)\\ \; & =\sum_{l=0}^{L-1}\alpha_{l}\frac{\sin\frac{\pi \left(t-\tau_{l}\right)}{T_{S}}}{\frac{\pi \left(t-\tau_{l}\right)}{T_{S}}}\frac{\cos\frac{\beta \pi \left(t-\tau_{l}\right)}{T_{S}}}{1-{\left(\frac{2\beta \left(t-\tau_{l}\right)}{T_{S}}\right)}^{2}}. \end{array}$$

Thus, the output signal y(t) is:

(19) $$\begin{array}{cc} y(t) & =s(t)*R(t)+W(t)\\ \; & =\sum_{m=-\infty }^{\infty }s\left(m\right)R\left(t-m\right)+W\left(t\right), \end{array}$$

where s(m)∈{+1,−1} represents bipolar information symbols with equal probability. The sampled y(t) can be calculated by:

(20) $$\begin{array}{cc} y(n)= & \sum_{m=-\infty }^{\infty }s\left(m\right)R\left(n-m\right)+W\left(n\right)\\ = & \underset{\mathrm{Expected}\;\mathrm{signal}\;}{\underbrace{s\left(n\right)R\left(0\right)}}+\underset{\mathrm{Intersymbol}\;\mathrm{interference}\;}{\underbrace{\underset{m=-\infty }{\sum_{m\neq n}^{m=\infty }}s\left(m\right)R\left(n-m\right)}}+\underset{\mathrm{Noise}\;}{\underbrace{W(n)}}. \end{array}$$

In Equation (20), the first term contains the expected symbol s(n) with m=n. The second term is the ISI from the other symbol s(m) with m≠n. Different from CBWCS with two ISI sources, the method proposed in this paper satisfies Nyquist ISI criteria, so only one ISI source caused by multi-path propagation needs to be considered.

To eliminate ISI caused by multi-path propagation, we take the second term of Equation (20) as the optimal threshold sequence $\theta {\left(n\right)}_{opt}$ [21]:

(21) $$\theta {\left(n\right)}_{opt}=\sum_{\begin{array}{c} m\neq n\\ m=-\infty \end{array}}^{\infty }s\left(m\right)R\left(n-m\right),$$

where R(n−m) can be calculated if the channel parameters $\tau_{l}$ and $\alpha_{l}$ are known. Substituting Equation (21) into Equation (17), the calculated threshold is used in the symbol decision stage to relieve ISI. Now, signal transmission is only effected by Gaussian white noise.

However, in order to obtain $\theta {\left(n\right)}_{opt}$ in Equation (21), two parts of symbols are needed: the past symbols s(m)(m<n) and the future symbols s(m)(m>n). The past symbols can be decoded directly, while the future symbols are not available. Thus, a suboptimal threshold with only the past symbols is described as:

(22) $$\theta {\left(n\right)}_{subopt}=\sum_{\begin{array}{c} m=-\infty \end{array}}^{n-1}s\left(m\right)R\left(n-m\right).$$

### 4.3. Performance Analysis

In this section, the BER performance and computational complexity of the proposed scheme are analyzed.

#### 4.3.1. BER Performance Analysis

- AWGN channel

**Figure 7:**
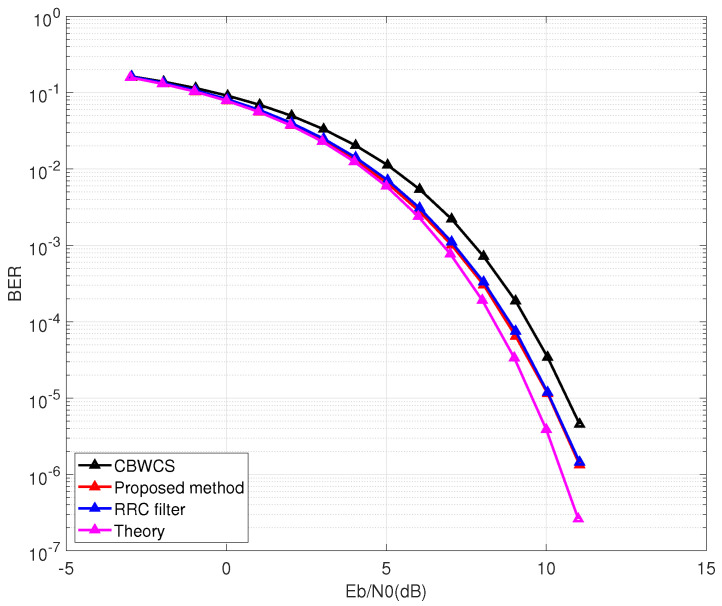
Bit error rate (BER) comparison under additive white Gaussian noise (AWGN) wireless channel.

For an AWGN channel (L=1), τ=0 and γ=0. The simulation results of CBWCS [21], the proposed method, conventional RRC filter, and the theoretical BER of BPSK under an AWGN channel are presented in Figure 7. In this simulation, for fair comparison, the threshold θ(n) was 0, and any channel equalization methods were not implemented at receiver. In all of the following simulations, the oversampling rate $N_{o}$ was 10, and the tap number of filter $N_{f}$ was 6. More than 100,000 trials were performed on average.

From Figure 7, since the proposed shaping filter is designed without ISI, the chaotic waveform can achieve the same BER performance as an RRC filter. CBWCS has the worst BER performance because of ISI caused by the chaotic filter itself.

- Multi-path channel

For a multi-path channel, we assessed the performance under two conditions: a two-path channel (L=2), $\tau_{0}=0$, $\tau_{1}=1$, γ=0.6, and a three-path channel (L=3), $\tau_{0}=0$, $\tau_{1}=1$, $\tau_{2}=2$, γ=0.6.

**Figure 8:**
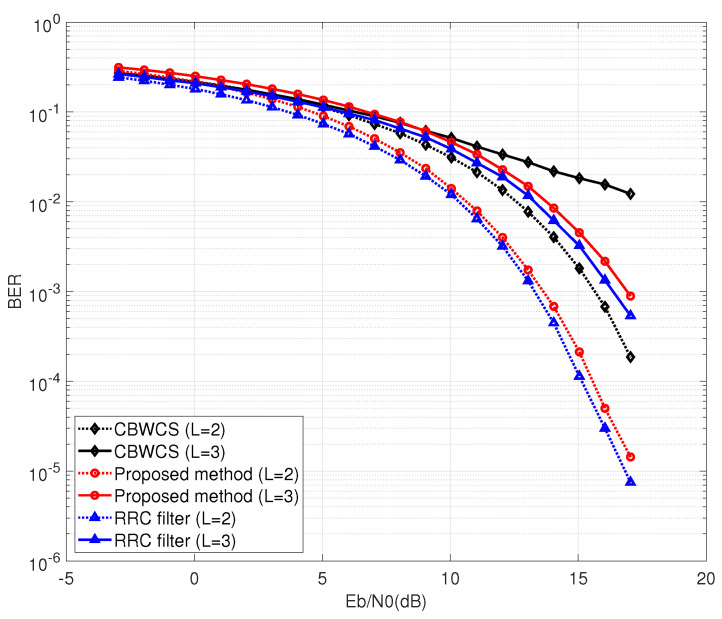
BER comparison under multi-path wireless channel with θ(n)=0.

First, we discuss the case with θ(n)=0 and no channel equalization. The BER performance comparison between CBWCS, the proposed method, and the RRC filter is shown in Figure 8. The dotted lines and solid lines indicate the performance under a two-path channel and a three-path channel, respectively. From Figure 8, it can be seen that ISI caused by multi-path propagation leads to worse BER performance compared with the AWGN channel. The RRC filter and the proposed method have similar BER performance, while CBWCS not only has ISI itself but is also effected by ISI due to multi-path propagation, so it has the worst BER performance. When BER = $10^{-3}$, the proposed method outperforms CBWCS by more than 2 dB under a two-path channel.

**Figure 9:**
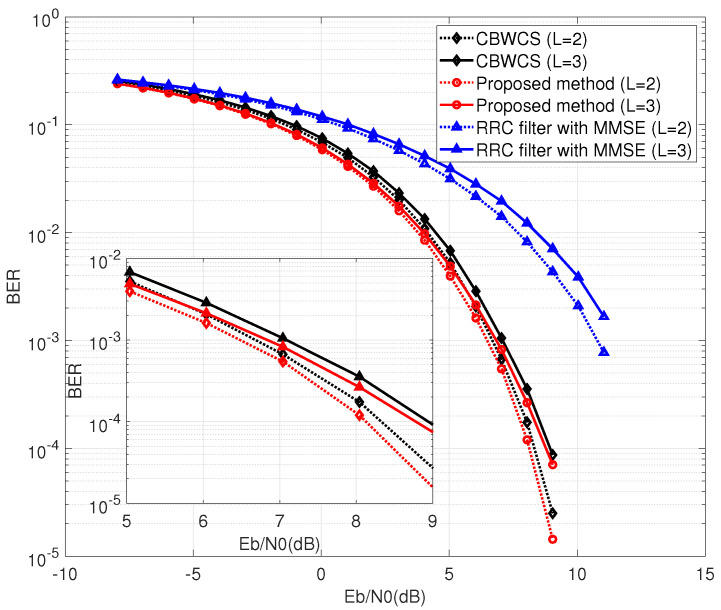
BER comparison under static multi-path wireless channel with optimal threshold.

Next, we implemented the calculated threshold in symbol detection. Figure 9 shows the comparison results of CBWCS, the proposed method, and the RRC filter with the optimal threshold (Equation (21)). For the conventional RRC-based communication system, a minimum mean square error (MMSE) equalizer is used for channel equalization. For BER = $10^{-3}$, the proposed method has about 0.2 dB and 0.25 dB gain under two-path and three-path channels compared with CBWCS, respectively, because the ISI, which is not completely eliminated, effects the accuracy of CBWCS. The gap between the chaotic waveform without channel equalization and the traditional non-chaotic waveform using linear equalization shows that the threshold can be implemented without amplifying the channel noise to combat multi-path effects, because the Lyapunov exponent of the chaotic waveform stays constant after passing through the wireless channel.

**Figure 10:**
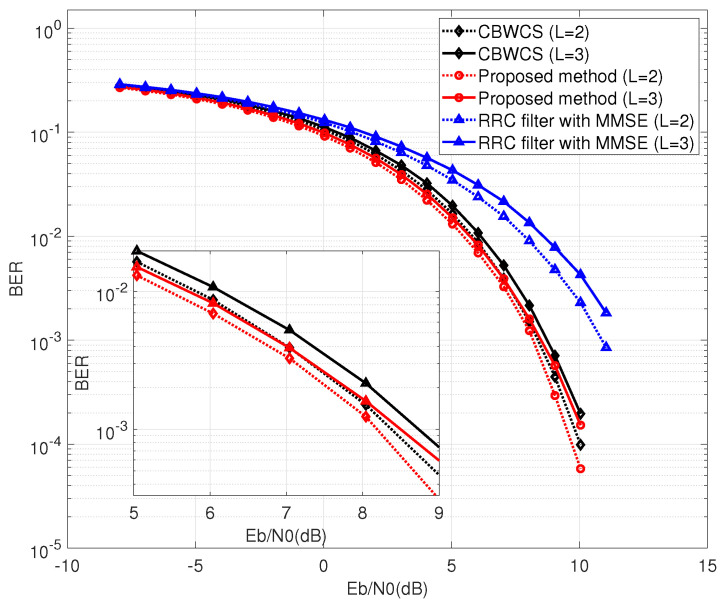
BER comparison under static multi-path wireless channel with suboptimal threshold.

Figure 10 shows BER performance with the suboptimal threshold (Equation (22)). Similar to Figure 9, the performance of the two-path channel generally outperforms that of the three-path channel. When BER is $10^{-3}$, the BER of the suboptimal threshold is about 1.7 dB worse than the optimal threshold.

**Figure 11:**
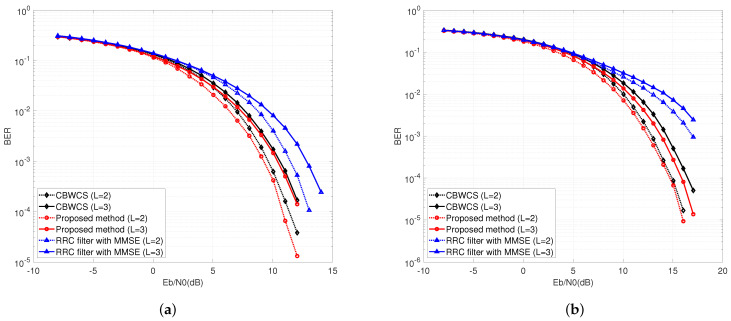
BER comparison under time-varying multi-path wireless channel with suboptimal threshold for (**a**) binary-phase shift keying (BPSK) and (**b**) 16 quadrature amplitude modulation (QAM).

All these analyses are based on the premise that the state of the channel, including decay and delay, are constant. Thus, for all frames, a perfect channel knowledge assumption is employed for threshold calculation. However, in an actual environment, the channel state of each frame will change. We assume that the channel parameter $\tau_{L}$ is invariant in one frame and uniformly distributed in the range of [0.3,0.9] in different frames of both the two-path channel and three-path channel. In this simulation, 1024 bits in one frame are divided into two parts: 896 data bits and 128 pilot bits. Pilot bits are used to carry out the least squares (LS) algorithm for channel estimation. Figure 11a shows the comparison of different methods under this quasi-static channel with the suboptimal threshold, where the BER performance deteriorates due to the inaccurate estimation of channel state information. However, the proposed threshold calculation method is still 2.2 dB better than traditional MMSE equalization.

In addition to BPSK, the proposed method can also be used in high-order modulation. Figure 11b shows the comparison of different methods for 16QAM, where BER performance degrades more than 3 dB compared with BPSK due to the distance between constellation points being reduced. The proposed method outperforms the CBWCS and RRC filters by 0.4 dB and 0.56 dB for BER = $10^{-3}$, respectively.

#### 4.3.2. Computational Complexity Analysis

In this subsection, we study the computational complexity of different wireless communication systems in terms of floating-point operations (flops). A flop is defined as one addition, subtraction, multiplication, or division of two floating point numbers, a comparison, and the usually accompanying fetch and store [27]. The flop count can be obtained by simply adding up all of the described operations. With this definition, one complex addition and one complex multiplication can be viewed as 2 and 6 flops, respectively.

The main differences between the proposed system and the traditional non-chaotic system are the pulse-shaping filter, corresponding matched filter and symbol decision algorithm. Thus, the number of flops of these three parts are discussed and shown in Table 1. $N_{s}$, $N_{o}$, $N_{f}$ and *L* are the length of the symbol, the oversampling rate, the tap number of the filter, and the multi-path number, respectively. The cost of executing pulse shaping for one symbol is $N_{f}$ multiplications and $N_{f}-1$ adds. Therefore, by omitting the lower-order terms, $2N_{o}N_{f}$ flops are required to carry out pulse shaping after oversampling with the sampling frequency $N_{o}$ for all three methods. Matched filtering has the same computational complexity as pulse shaping. To decode one symbol, in a non-chaotic communication system, the computational complexity of the decoding algorithm mainly caused by MMSE equalization is $24L^{3}$ flops [28]. In CBWCS, for each optimal threshold calculation, one convolution needs $38N_{s}$ mathematical operations, $N_{s}-1$ multiplications and $N_{s}-2$ additions, while in the proposed method, because the threshold has a simpler impulse response expression, the flops of one convolution are reduced to $17N_{s}$ mathematical operations, $N_{s}-1$ multiplications and $N_{s}-2$ additions. Multiplying by the multi-path number and omitting the lower-order terms, the computational complexity of threshold calculation in CBWCS and the proposed system are $40N_{s}L$ flops and 19$N_{s}L$ flops, respectively. The reduction of 52.5% flops in threshold calculation means that the proposed method has a performance advantage in terms of overall computational complexity.

In threshold calculation, because the response of R(t) will rapidly decay to zero, $\theta_{opt}$ only depends on the current and future $N_{p}$ non-zero coefficients of R(t). In CBWCS, for $N_{p}=5$, the requirement is satisfied, and in the proposed method, $N_{p}=6$. Thus, for L≥3, the RRC with MMSE has the highest computational complexity, while the proposed method has the key advantages of involving less computational complexity and a simpler structure of the decision threshold.

## 5. Conclusions

In this paper, a novel chaotic pulse-shaping filter and matched filter from RRC division are proposed. The unequal RRC division method provides the possibility to generate chaotic waveforms while ensuring no ISI. Based on this, a wireless communication system architecture using generated chaotic waveforms is developed, where a chaotic pulse-shaping filter and corresponding matched filter are used to implement encoding at the transmitter and maximize the SNR during decoding at the receiver, respectively. In order to guarantee that the information can be retrieved from the received signal, a continuous-time chaotic waveform is proved to be topologically conjugate to the bi-polar information. Simulation results in a single-path channel and multi-path channel with a time delay validate its effectiveness and superiority. Under an AWGN single-path channel, the proposed chaotic waveform can achieve the same BER performance as an RRC filter, which is very close to the lower bound of the BER. Under a multi-path channel, the proposed method is demonstrated to be 2 dB better than CBWCS when the threshold is zero. In the case with optimal and suboptimal thresholds, compared with CBWCS, the proposed scheme reduces computational complexity by 52.5% without effecting the BER performance.

## Figures and Tables

**Table 1:** Computational complexity comparison.

Method	Pulse Shaping	Matched Filtering	Decoding
Root-raised-cosine (RRC) with minimum mean square error (MMSE)	$2N_{o}N_{f}$	$2N_{o}N_{f}$	$24L^{3}$
Chaotic baseband wireless communication system (CBWCS)	$2N_{o}N_{f}$	$2N_{o}N_{f}$	$40N_{s}L$
Proposed method	$2N_{o}N_{f}$	$2N_{o}N_{f}$	$19N_{s}L$

## Data Availability

Not applicable.
